# Population Structure and Historical Demography of the Oriental River Prawn (*Macrobrachium nipponense*) in Taiwan

**DOI:** 10.1371/journal.pone.0145927

**Published:** 2015-12-30

**Authors:** Po-Cheng Chen, Chun-Han Shih, Ta-Jen Chu, Daryi Wang, Ying-Chou Lee, Tzong-Der Tzeng

**Affiliations:** 1 Institute of Fisheries Science, College of Life Science, National Taiwan University, Taipei, 106, Taiwan; 2 Department of Leisure Management, Tungnan University, New Taipei City, 222, Taiwan; 3 Department of Leisure and Recreation Management, Chung Hua University, Hsin Chu, 300, Taiwan; 4 Biodiversity Research Center, Academia Sinica, Taipei, 115, Taiwan; 5 Department of Leisure, Recreation and Tourism Management, Shu-Te University, Kaohsiung, 824, Taiwan; Shanghai Ocean University, CHINA

## Abstract

The oriental river prawn (*Macrobrachium nipponense*) is a non-obligatory amphidromous prawn, and it has a wide distribution covering almost the entire Taiwan. Mitochondrial DNA fragment sequences of the cytochrome oxidase subunit I (COI) and 16S rRNA were combined and used to elucidate the population structure and historical demography of oriental river prawn in Taiwan. A total of 202 individuals from six reservoirs and three estuaries were separately collected. Nucleotide diversity (*π*) of all populations was 0.01217, with values ranging from 0.00188 (Shihmen Reservoir, SMR, northern Taiwan) to 0.01425 (Mingte Reservoir, MTR, west-central Taiwan). All 76 haplotypes were divided into 2 lineages: lineage A included individuals from all sampling areas except SMR, and lineage B included specimens from all sampling locations except Chengching Lake Reservoir (CLR) and Liyu Lake Reservoir (LLR). All *F*_ST_ values among nine populations were significantly different except the one between Jhonggang River Estuary (JGE, west-central Taiwan) and Kaoping River Estuary (KPE, southern Taiwan). UPGMA tree of nine populations showed two main groups: the first group included the SMR and Tamsui River Estuary (TSE) (both located northern Taiwan), and the second one included the other seven populations (west-central, southern and eastern Taiwan). Demographic analyses implied a population expansion occurred during the recent history of the species. The dispersal route of this species might be from China to west-central and west-southern Taiwan, and then the part individuals belonging to lineage A and B dispersed southerly and northerly, respectively. And then part individuals in west-central Taiwan fell back to and stay at estuaries as the sea level rose about 18,000 years ago.

## Introduction

The present population genetic structure of a species may be fully interpreted if the influence of historical events and the complex interactions of biology, geography, and climatic shifts were considered [1]. Stern climatic shifts can create great changes in species’ geographical distribution and abundance, and the advent of DNA technology provides proper markers to reflect the genetic effects under the influence of these changes [1, 2]. Mitochondrial DNA (mtDNA) is highly polymorphic, and it has been used for assessing population genetic structure, phylogeographical history to provide demographic inferences in relation to population dynamics [2]. Moreover, the effective population size of using mtDNA is smaller than that of nuclear DNA due to its haploid structure and maternal inheritance [3].

There were drastic changes in areas and configurations in marginal seas of the Western Pacific during the late Quaternary glacial cycles [4]. For example, in the Last Glacial Maximum, the sea level came to its lowest, at about 130 m below the present sea level, which result in extensive areas of the Northeast and Southeast Asian continental shelves became land bridges connecting the islands of Japan and Taiwan to the mainland of Asia [5, 6]. As a result, the present genetic structures of populations in the Western Pacific had been greatly influenced throughout Pleistocene ice ages. The cyclic separation and rejoining of Taiwan with the mainland China caused by glacial sea-level changes provided opportunities for taxa dispersing between these two areas [7, 8].

The spatially disconnected river systems form unique natural geographical constraints on freshwater fauna [2]. The dispersal of freshwater species between river drainages is normally extremely limited and dependent on direct connections between drainage basins, such as drainage re-arrangements, short-term connections between drainages, and sea level changes [9]. During the glacial period, many rivers which were separated by the sea became connected to one another. These connected waterways could facilitate the migration of freshwater fauna between river systems [10]. Accordingly, geological events have greatly influenced endemism, and the population structure of freshwater fauna.

The oriental river prawn (*Macrobrachium nipponense*) distributes broadly over the East Asian regions (China, Japan, Korea, Vietnam, Myanmar and Taiwan) [11, 12], and has been introduced in Singapore, Philippines [13], Uzbekistan [14], southern Iraq [15] and Iran [16]. In Taiwan, it has a wide distribution covering almost the entire island. This species was suggested to be originated in mainland China and then dispersed to Taiwan through the land bridges between Taiwan and China during Pleistocene [17]. Oriental river prawn is a non-obligatory amphidromous prawn [18, 19]. A number of oriental river prawns remain in the estuaries (river mouths) to complete their life cycle, but some populations are found in coastal or inland freshwater lakes, resulting from a shift in its habitat from estuaries to inland freshwaters [20, 21]. The gene flow between populations in inland freshwater is obviously lower than the one between populations in estuaries, and thus phylogeographical analyses of populations in inland freshwater are more easy inference on the association between biotic and geological factors [22, 23].

Only one paper analyzed morphological characters in an attempt to determine the population structure of this species in Taiwan [24]. The findings suggest that these populations clustered into estuarine group, west-south-reservoir group, and north-reservoir group. However, variations in morphological characters can be affected by both genetic and environmental factors, so that discrimination of populations based on morphological variations must be verified by genetic evidence to confirm that the variations reflect the true degree of reproductive isolation rather than environmental isolation [25]. Furthermore, the historical demography of oriental river prawn in Taiwan is still unknown. In this study, we used mtDNA fragment sequences of cytochrome oxidase subunit I (COI) and 16S rRNA gene to reveal the population structure and historical demography of the oriental river prawn populations at the six reservoirs and three estuaries in Taiwan.

## Materials and Methods

### Sample collection

**Fig 1:**
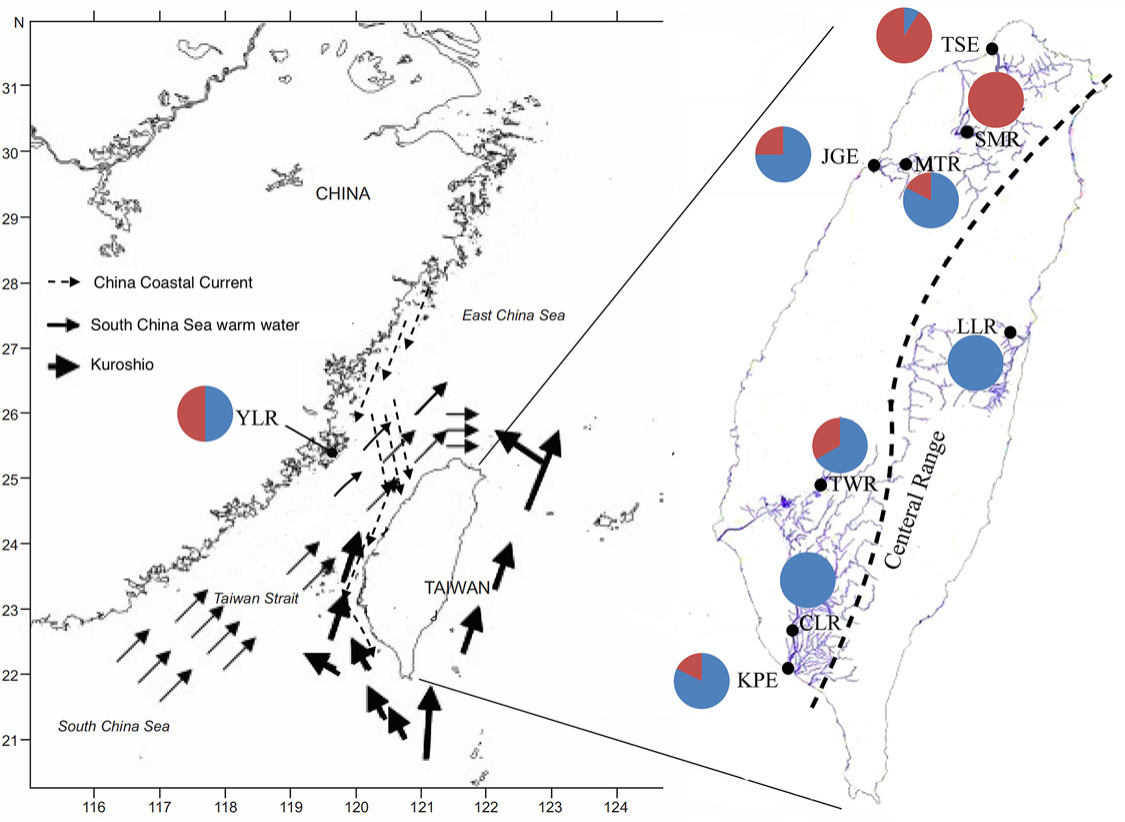
Sampling localities and haplotypes frequencies of *Macrobrachium nipponense* in Taiwan. Numbers of lineage A and B in each sampling site were also shown in Table 1.

All samples were collected in open and public waters, and thus specific permission is not necessary. The specimens were collected by five bait traps each reservoir and each estuary from the evening 17:00 to next morning 7:00 during successive 3 days in winter season, from December 2013 to January 2014. A total of 202 individuals were separately collected from six reservoirs, Shihmen Reservoir (SMR, northern Taiwan), Mingte Reservoir (MTR, west-central Taiwan), Tsengwen Reservoir (TWR, southwestern Taiwan), Chengching Lake Reservoir (CLR, southern Taiwan), Liyu Lake Reservoir (LLR, eastern Taiwan) and Yangming Lake Reservoir (YLR, southeastern China), and three estuaries, Tamsui River Estuary (TSE, northern Taiwan), Jhonggang River Estuary (JGE, west-central Taiwan) and Kaoping River Estuary (KPE, southern Taiwan) (Fig 1; Table 1). The specimens were immediately iced or frozen after capture and kept at -75°C for DNA extraction.

**Table 1:** Codes of sampling sites, sample size (*n*), number of haplotypes (*n*_*h*_), gene diversity (*h*), and nucleotide diversity (*π*), Tajima’s *D*, and Fu’s *Fs* statistics in 9 populations of *Macrobrachium nipponense* in Taiwan.

Code	Sample site	*n*	*n_h_*	Lineage A	Lineage B	*h*	π	Tajima's *D*	Fu's *Fs*
SMR	Shihmen Reservoir	26	6	0	26	0.658 ± 0.062	0.00188 ± 0.00060	-1.409	-0.675
MTR	Mingte Reservoir	24	18	19	5	0.964 ± 0.025	0.01425 ± 0.00258	-1.296	-3.583^*^
TWR	Tsengwen Reservoir	24	16	16	8	0.938 ± 0.034	0.01365 ± 0.00260	-1.139	-1.882
YLR	Yangmin Lake Reservoir	28	12	14	14	0.931 ± 0.022	0.00950 ± 0.00069	1.181	0.373
CLR	Chengching Lake Reservoir	22	11	22	0	0.883 ± 0.053	0.00671 ± 0.00202	-1.794	-1.103
LLR	Liyu Lake Reservoir	19	14	19	0	0.959 ± 0.031	0.00524 ± 0.00091	-0.905	-6.727^**^
TSE	Tamsui River Estuary	22	7	2	20	0.671 ± 0.094	0.00502 ± 0.00139	-1.155	1.128
JGE	Jhonggang River Estuary	20	7	15	5	0.816 ± 0.050	0.00920 ± 0.00085	1.366	3.166
KPE	Kaoping River Estuary	17	5	14	3	0.801 ± 0.056	0.00880 ± 0.00122	0.947	4.946
Lineage A		121	55			0.966 ± 0.007	0.00834 ± 0.00058	-1.783^*^	-36.553^**^
Lineage B		81	21			0.733 ± 0.049	0.00450 ± 0.00110	-2.489^**^	-6.249^**^
Total		202	76			0.945 ± 0.010	0.01217 ± 0.00054	-1.773^*^	-42.78^**^

^*^ *P* < 0.05

^**^ *P* < 0.01.

### DNA extraction, amplification and sequencing

Total genomic DNA was extracted from abdominal muscle tissue following the treatment of a standard proteinase K, phenol–chloroform extraction, and ethanol precipitation [26]. Two different fragments (16S rRNA and COI) in mtDNA were amplified and sequenced. The 16S rRNA and COI sequences were amplified using these 1471 (5’-CCT GTT TAN CAA AAA CAT-3’) and 1472 (5’-AGA TAG AAA CCA ACC TGG-3’) [27] and COI-F (TTT ATC TTC GGA GCG TGA GC) and COI-R (AGT TAT TCC TGG GGC TCG TAT G) [28] primers, respectively. Thermal cycling was performed on GeneAmp 2400 thermal cycler (Perkin-Elmer, Norwalk, CT, USA) and PCR conditions consisted of 39 cycles of denaturation at 95°C for 50 s, annealing at 50°C for 1 min, and extension at 72°C for 1.5 min. An initial denaturation step at 95°C for 5 min and a final extension holding at 72°C for 10 min were respectively included in the 1st and last cycles. PCR product was separated by electrophoresis on 1.5% agarose gels, purified with the Gene Clean II kit (Bio101, Vista, CA, USA), and sequenced on an ABI 377 DNA sequencer (Applied Biosystems, Inc.; Foster City, CA, USA).

### Sequence analyses

All sequences were aligned using MegAlign (DNASTAR, LaserGene, WI, USA). The number of variable and parsimony informative sites, base composition, and transition/transversion ratios, and haplotype diversity and nucleotide diversity [29] were calculated using DnaSP version 5.00 [30].

Part of sequences of the 16S rRNA and COI genes were concatenated in following analyses. There were used 16S and COI fragment in network methods. Phylogeographic analyses were carried out by the neighbor-joining (NJ) and maximum likelihood (ML) methods based on 16S rRNA and COI gene respectively by MEGA [31]. We used bootstrap analyses the NJ and ML methods with 1,000 replicates to evaluate support for phylogenetic relationships. The optimal substitution model for DNA sequences were determined by using MEGA 6. A network of haplotypes was also constructed using the median-joining method [32] in Network vers. 4.6.1.3 available at www.fluxus-engineering.com. Rough dates of population expansion were estimated with the formula τ = 2μ*T* [33], where *T* is the time since expansion, τ is the expansion time, and 2 μ is μ (the mutation rate) x generation time x number of bases sequenced. The average divergence rate of 1.17–1.66% per million years and a generation time of one year were used here [34].

To examine the genetic differentiation between any two of the populations, pair-wise *F*_ST_ statistics were estimated by ARLEQUIN 3.5 [35]. The dendrogram of nine sampling sites was also constructed using the unweighted pair-group method with arithmetic means (UPGMA) based on the *F*_ST_ values. The population structure was also assessed by analysis of molecular variance (AMOVA; [36] in ARLEQUIN). Various groupings of these populations were suggested by UPGMA tree of these nine populations. The grouping that revealed the maximal value of Φ_CT_ and significantly differed from a random organization of similar groupings was assumed to represent the most-probable geographic subdivisions [37]. The significance test of statistical result was evaluated by permutations test with 10,000 random permutations.

Tajima’s *D* [38] was used to check for deviations from neutrality, indicating whether population expansion had occurred in the past. Fu’s *Fs* test [39] was also carried out to assess evidence for population expansion using DnaSP. In addition, population expansion was also investigated with mismatch analysis to examine the frequency distributions of nucleotide difference as a function of frequency by DnaSP.

## Results

In total, 202 specimens were sequenced. The size of the 16S rRNA fragment was 421 bp, with 79 variable sites and 34 parsimony informative sites, resulting in 34 unique haplotypes (S2 Dataset). All sequences were deposited in GenBank, with accession numbers KU235597—KU235798. No gaps were detected, and the numbers of transitions outnumbered transversions in all comparisons is approximately 1.0-fold. The frequency of nucleotide composition showed an AT bias (with G + C contents of 58.9%). The size of the COI fragment was 371 bp; there were 43 variable sites and 23 parsimony informative sites, resulting in 38 unique haplotypes (S3 Dataset). All sequences were deposited in GenBank, with accession numbers KU235799—KU236000. The numbers of transitions outnumbered transversions in all comparisons is 1.04-fold on average. The frequency of nucleotide composition showed an AT bias (with G + C contents of 39.3%).

Following results were obtained by analyzing the combined sequences of the 16S rRNA and COI genes (S1 Dataset). The haplotype diversity (*h*) of all nine populations was 0.945, with values ranged from 0.658 (SMR) to0.964 (MTR) (Table 1). The nucleotide diversity (*π*) of total populations was 0.01217, with values ranged from 0.00188 (SMR) to 0.01425 (MTR) (Table 1). A total of 76 haplotypes was detected in 202 specimens. The most common allele was shared by 40 individuals from these populations of TSE (12), SMR (10), YLR (5), TWR (5), JGE (5) and KPE (3). The second common allele was shared by 13 individuals from the populations of CLR (7), TWR (4), TSE (1) and JGE (1). Two different alleles were separately shared by 12 individuals from the populations of SMR (12) and KPE (6) and JGE (6).

**Fig 2:**
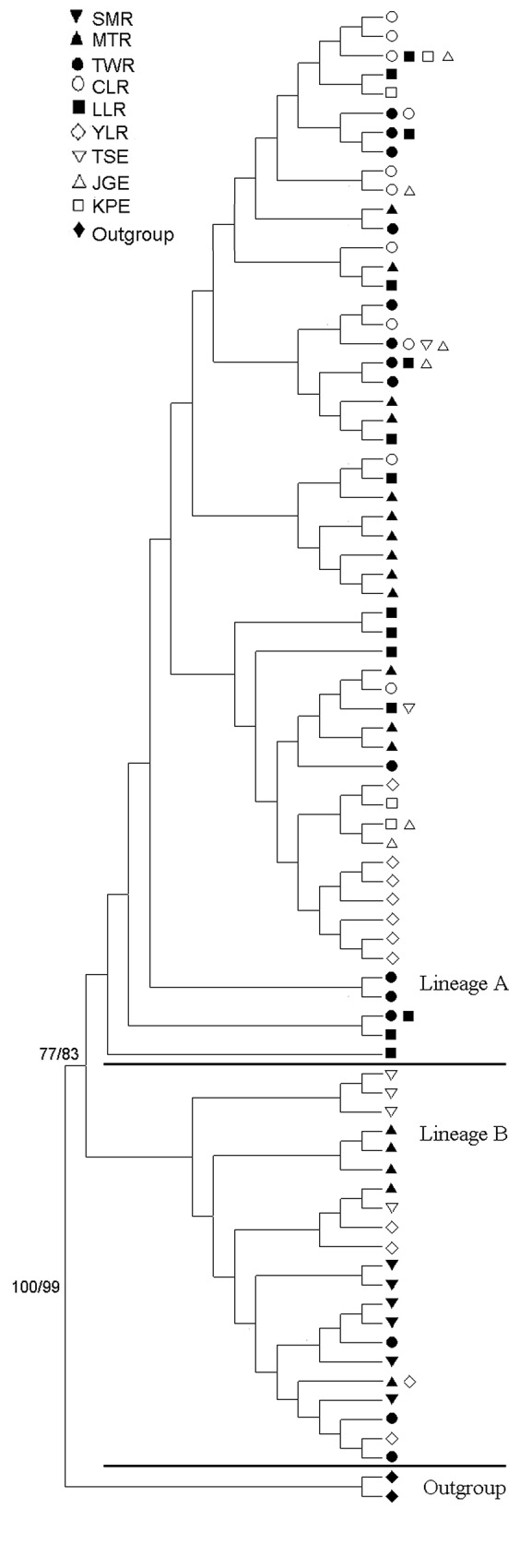
Neighbor-joining (NJ) tree based on mtDNA 16S + COI sequences with bootstrap values (NJ/ML, respectively) shown adjacent to corresponding two lineages for *Macrobrachium nipponense*. Number at the nodes indicate bootstrap values (expressed as percentage) with 1,000 replicates.

**Fig 3:**
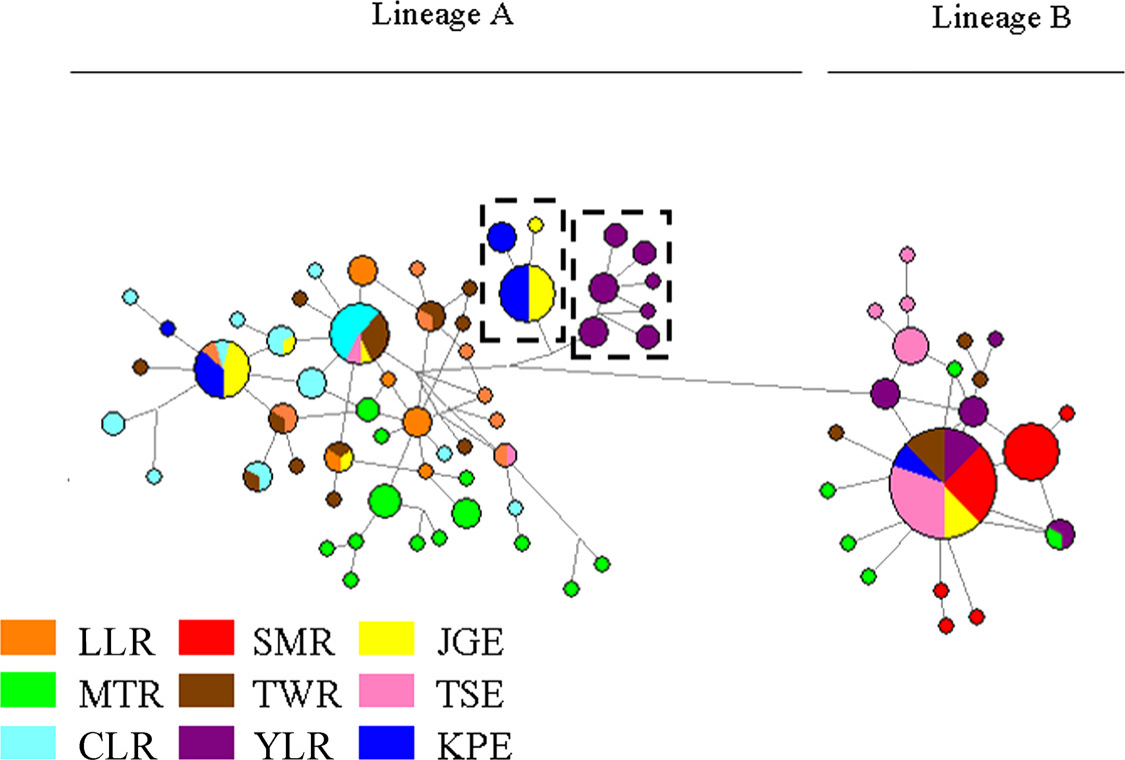
The haplotype network of *Macrobrachium nipponense* in all sampling sites.

The best-fitting model explaining our data was T92 model. This model was used for NJ and ML reconstruction and AMOVA analyses. Two individuals of *Macrobrachium asperulum* were also analyzed in NJ and ML reconstruction as outgroups. Phylogenetic tree of all haplotypes is shown in Fig 2. Bootstrap values of main node are 77 and 83 for neighbor join and Maximum Likelihood trees, respectively. All haplotypes were divided into two distinct lineages (A and B). The network for all specimens (Fig 3) supported the result obtained from the phylogenetic tree. Two sub-lineages might be found in lineage A in the network. The first sub-lineage only included individuals from YLR population, and the second one comprised the specimens from KPE and JGE populations. These two sub-lineages were also found at the basal node of the lineage A of phylogenetic tree. The distribution of lineage A and B specimens for different populations are also shown in Fig 1 and Table 1. Both individuals from lineage A and B could be found in all sampling sites except SMR, CLR and LLR. Both CLR and LLR populations only included individuals from lineage A, but SMR comprised ones from lineage B. Haplotype diversities (*h*) of lineage A and lineage B were 0.966 and 0.733, respectively. Nucleotide diversities (π) of lineage A was 0.00834, and the lineage B was 0.00450 (Table 1). The τ values of lineage A and lineage B were 5.215/2u and 0.932/2u generations, respectively. The average divergence rate of 1.42% / myr and a generation time of 1 year were used to calculate the time of expansion. The estimated time of expansion for lineage A was 257,902 years ago. For lineage B, the estimate was 41,435 years ago.

**Fig 4:**
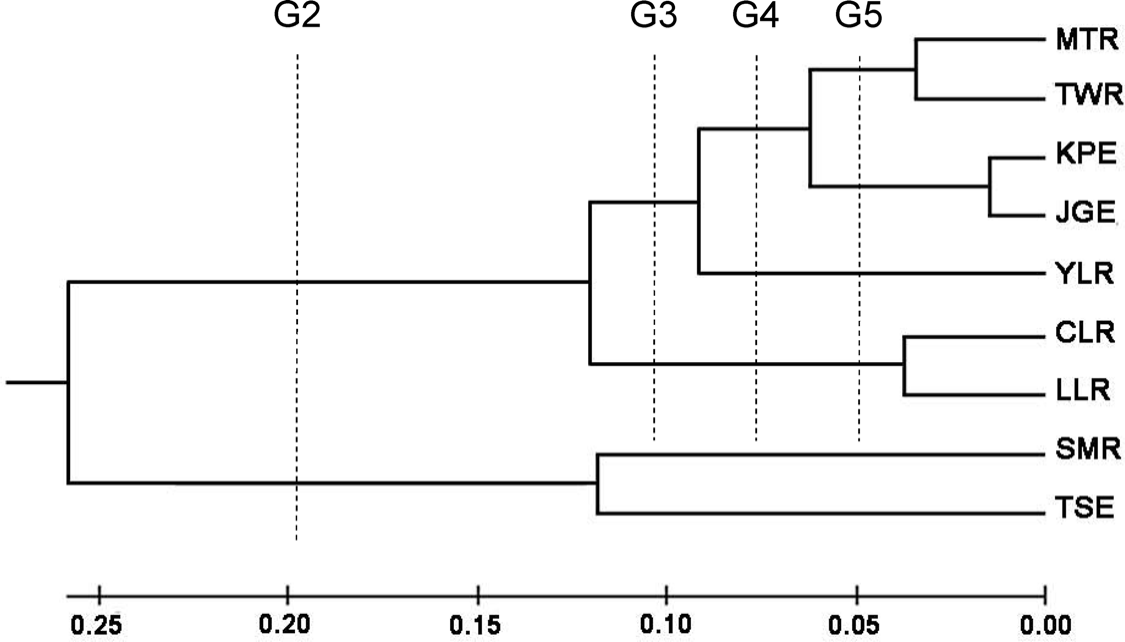
UPGMA tree showing relationships among the 9 sampling sites.

All *F*_ST_ values among nine populations were significant except the one between KPE and JGE (Table 2). The UPGMA tree of these nine sampling areas was computed after allowing a 1000 replicate bootstrap test using the same program (Fig 4). These nine populations could be divided into two main groups: the first group included the SMR and TSE, and the second one included the other seven populations. The second group may be further divided into four subgroups; the first subgroup included the CLR and LLR populations; the second subgroup included the YLR; the third subgroup included KPE and JGE; the fourth subgroup included MTR and TWR.

**Table 2:** Matrix of pairwise *FST* (below diagonal) and *P* values (above diagonal) among 9 populations of *Macrobrachium nipponense* in Taiwan.

	SMR	MTR	TWR	YLR	CLR	LLR	TSE	JGE	KPE
SMR	-	0.000	0.000	0.000	0.000	0.000	0.000	0.000	0.000
MTR	0.553	-	0.000	0.013	0.000	0.000	0.000	0.000	0.000
TWR	0.445	0.069	-	0.001	0.001	0.008	0.000	0.025	0.030
YLR	0.393	0.236	0.158	-	0.000	0.000	0.002	0.002	0.000
CLR	0.781	0.172	0.135	0.449	-	0.006	0.000	0.000	0.000
LLR	0.801	0.103	0.113	0.427	0.075	-	0.000	0.000	0.000
TSE	0.236	0.416	0.263	0.257	0.631	0.653	-	0.000	0.000
JGE	0.589	0.153	0.163	0.066	0.205	0.234	0.371	-	**0.682**
KPE	0.636	0.190	0.085	0.177	0.267	0.300	0.000	**0.030**	-

Five different groupings for nine populations were suggested by UPGMA trees. The results of AMOVA are shown in Table 3. The AMOVA for nine populations yielded a significant F_ST_ value of 0.3434, indicating that at least one of the pair-wise populations reveals significant heterogeneity. Significant values of Φ_CT_ were observed in all groupings. The highest Φ_CT_ values (0.3392) were found in the grouping 2, and supported these nine populations could be divided into two main groups: the first group included the SMR and TSE, and the second one included the other seven populations. Significant Φ_CT_ values were also found in different groupings, indicating that an additional genetic discontinuity may also have occurred among populations.

**Table 3:** AMOVA results for 9 populations of *Macrobrachium nipponense* in Taiwan.

Grouping		Source of variation	Percentage of variation	Φ-Statistics	Significance
One group	Group 1 {SMR, MTR, TWR, YLR, CLR, LLR, TSE, JGE, KPE}	AP	34.34	Φ_ST_ = 0.3434	^***^
		WP	65.66		
Two groups	Group 1 {MTR, TWR, YLR, CLR, LLR, KPE, JGE}	AG	33.92	Φ_CT_ = 0.3392	^**^
	Group 2 {SMR, TSE}	AP/WG	13.59	Φ_SC_ = 0.4751	^***^
		WP	52.49	Φ_ST_ = 0.4027	^***^
Three groups	Group 1 {CLR, LLR}	AG	30.37	Φ_CT_ = 0.3037	^***^
	Group 2 {SMR, TSE}	AP/WG	10.66	Φ_SC_ = 0.1531	^***^
	Group 3 {TWR, MTR, YLR, KPE, JGE}	WP	58.98	Φ_ST_ = 0.4103	^***^
Four groups	Group 1 {CLR, LLR}	AG	30.42	Φ_CT_ = 0.3042	^***^
	Group 2 {SMR, TSE}	AP/WG	7.99	Φ_SC_ = 0.1148	^***^
	Group 3 {TWR, MTR, KPE, JGE}	WP	61.59	Φ_ST_ = 0.3841	^***^
	Group 4 {YLR}				
Five groups	Group 1 {CLR, LLR}	AG	31.81	Φ_CT_ = 0.3181	^***^
	Group 2 {SMR, TSE}	AP/WG	4.74	Φ_SC_ = 0.0709	^***^
	Group 3 {KPE, JGE}	WP	63.46	Φ_ST_ = 0.3042	^***^
	Group 4 {TWR, MTR}				
	Group 5 {YLR}				

AG is the among-group component of variance; AP/WG is the among-populations/within-group component of variance, and WP is the within-population component of variance.

^**^ *P* < 0.01

^***^ *P* < 0.001 by the permutation test.

**Fig 5:**
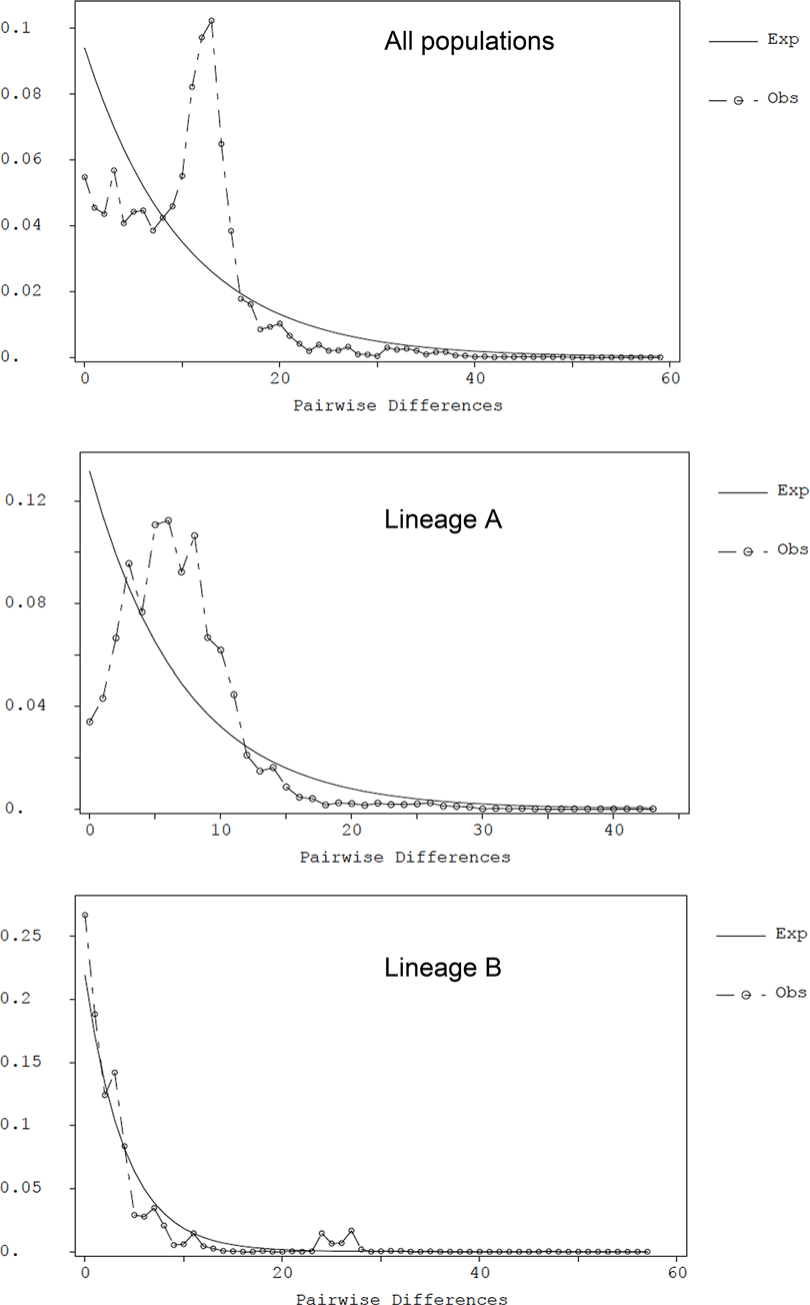
The observed pair-wise differences and the expected mismatch distributions under sudden expansion model of oriental river prawn. (a) All populations, (b) Lineage A, (c) Lineage B.

No significant Tajima’s *D* found in this population (Table 1). However, Tajima’s *D* values were significant for each lineage and for when all populations were combined. The Fu’s *F*s tests were significant for MTR and LLR populations (Table 1). The significant Fu’s *F*s value was also obtained when all populations were combined. The significant Fu’s *F*s values were found for lineage A and lineage B. The mismatch distribution of all specimens was bimodal (Fig 5A), with one mode corresponding to the number of differences within the lineages and the other one to differences between the two lineages. Separate analyses of lineage A and B in both cases yielded a unimodal distribution, which did not significantly differ (as measured by the sum of the squared deviation; *P* > 0.05) from that predicted by the growth expansion model (Fig 5B and 5C).

## Discussion

The spatially disconnected river systems form geographical barrier on freshwater fauna, and seldom or no gene flow occurs between populations in different rivers [2]. Obviously, all *F*_ST_ values among 5 inland populations were significant difference (Table 2). However, no significant genetic difference was found between both KPE and JGE estuarine populations, but the TSE population was significantly different from KPE and JGE. The migratory distance of the oriental river prawn in estuary was limited [40, 21]. Therefore, the dispersal of larvae often play important role in reducing variation between estuarine populations, and ocean currents is the main driving power in the dispersal of a species. For the oriental river prawn, it took 28 days for zoea larvae to metamorphose into juveniles [41], and that supplies a chance to yield major gene flow among estuarine populations. A portion of the Kuroshio Current flows into Taiwan Strait along the south-western coast of Taiwan, and the water masses of the South China Sea flow through the Taiwan Strait into the East China Sea (Fig 1). The three estuarine areas were primarily exchange overlaid by Kuroshio water masses and the water masses of the South China Sea. Therefore, gene flow from south to north generally occurs. A number of studies have shown the China coastal water flows across Taiwan Strait to the mid-west Taiwan and then flows south [42]. Therefore, this current cause the phenomenon of larvae in bank of western Taiwan (not including TSE) transported to south Taiwan [43, 44]. These currents may result in the exchange of larvae between both KPE and JGE and in homogeneity between them. However, unidirectional gene flow from south (KPE and JGE) to north (TSE) could not prevent from heterogeneity between TSE and the rest of two estuarine populations. The individuals of lineage A from KPE (14/17) and JGE (15/20) populations are apparently larger than ones in TSE (2/22), but the individuals of lineage B from in TSE (20/22) are obviously larger than ones in KPE (3/17) and JGE (5/20) (Table 1 and Fig 1). Only two individuals of lineage A were found in TSE population, and not shared with KPE and JGE populations. However, one haplotype in lineage B (including 40 specimens) was shared by three estuarine populations (Fig 3). Furthermore, we also found that SMR population only included individuals from lineage B. Individuals of lineage A may not adapt in northern Taiwan, and that may partly explain why the TSE population was significantly different from KPE and JGE. Individuals of lineage B were rare (KPE) or none (CLR) in southern Taiwan, and they may also have to face the adaption of environment, as like individuals of lineage A in northern Taiwan.

Although significant genetic differences were found in each pair of nine populations (Table 2) and the hierarchical AMOVA revealed that a significant genetic structure across all hierarchical levels existed among the nine populations were obtained, no obvious geographic division was found in genealogic reconstructions except YLR in lineage A (Fig 3). This outcome is evidently different from the outcome of a high degree of phylogeographically related genetic structure were often found in Taiwan [45, 4, 46–52]. This may be because the estimate of time of expansion for lineage A (257,902 yrs ago) or lineage B (41,435 yrs ago) was too short to form geographically unique clade.

The genealogic reconstructions of the oriental river prawn suggested that they can be consisted of two main lineages (Figs 2 and 3). The individuals in SMR (northern Taiwan) were only found in lineage B, and the specimens from MTR (west-central Taiwan), TWR (southwestern Taiwan), three estuarine populations (KPE, JGE and TSE) and YLR (near China) were separately included in lineage A or B, and the specimens in CLR (southern Taiwan) and LLR (eastern Taiwan) were only discovered in lineage A. Obviously, the west-central and southwestern Taiwan and coastal waters of Taiwan were mixing areas of lineages A and B of the oriental river prawn in Taiwan. The oriental river prawn originated from mainland China and dispersed to Taiwan, through the Pleistocene land bridges between Taiwan and mainland China, and its arrival time was apparently less than one million years ago [17]. Geological evidence indicates that land bridges connected the island to the Asian continent two to three times in the Pleistocene [53–55]. The estimated time could stand for the arrival time of this species from mainland China to Taiwan, and this time was in agreement with the one obtained by previous paper [17]. The nucleotide diversities (0.0083) and haplotype diversity (0.966) in the lineage A were significant higher than the ones (*π* = 0.0045; h = 0.733) in the lineage B. This suggested that lineage A was older than lineage B. The lineages A and B might originate from the same ancestor in mainland China, and then dispersed to Taiwan in different time. The lineage A moved to Taiwan earlier than lineage B, and the viewpoint was supported that haplotyps from YLR population has been formed unique sub-lineage in lineage A (Figs 2 and 3), but not developed unique sub-lineage in lineage B. The estimate of time of expansion for lineage A (257,902 yrs ago) and lineage B (41,435 yrs ago) also supported this point.

Theoretical studies have demonstrated that populations in long-term stable demographic equilibrium show a chaotic mismatch distribution, while recent rapid population expansions or bottlenecks translate into a unimodal (approximately Poisson) profile, with a steeper wave indicative of a smaller initial population before the expansion [33, 56]. An analysis of the demographic history of this prawn from the these two lineages seems to indicate that the lineage B displays a steeper wave, which is typical of a smaller initial population prior to the expansion or bottleneck (Fig 5) [33].

Populations with ancestral genotypes tend to preserve higher nucleotide and haplotype diversities because of long-term accumulation of mutations [57, 58, 47]. Most individuals in MTR formed various unique haplotype, not shared by other individuals from other sampling sites (Figs 2 and 3). This indicated MTR population had longer time to accumulate mutations than ones from different sampling sites in Taiwan. Furthermore, the greater nucleotide and haplotype diversities of MTR population indicated MTR population was ancestral population (Table 1). The traits of obvious higher nucleotide and haplotype diversities were also found in TWR population. However, some individuals in TWR formed various unique haplotype, but four (lineage B) and two (lineage A) individuals were separately involved in the most and third common alleles shared by other individuals from other sampling sites (Figs 2 and 3). Furthermore, MTR and TWR populations were clustered into the unique group (Fig 4), indicating very close relationship between the two populations. TWR population may also be ancestral population, but it had closer relationship with the other populations than MTR.

According to previous studies, the phylogeographical patterns of freshwater fishes usually reveal a close relationship between populations of the west-central and northern regions in Taiwan [7, 4, 59]. However, in the genealogic analyses (Figs 2, 3 and 4) showed that populations of the oriental river prawn of the west-central and southern regions were closely related as indicated by two freshwater fishes [47, 48] and one fresh prawn [52]. This may have been caused by different dispersal events and routes, through which species migrated from mainland China to Taiwan. This result is in agreement with the ones obtained from the *Acrossocheilus paradoxus* [47] and the *Macrobrachium asperulum* [52], but is different from the hypothesis of the northern and southern origins that freshwater fishes first immigrated into northern or southern Taiwan during glaciations, and then respectively dispersed southward or northward [7, 4, 47, 48]. And then the part individuals belonging to lineage A and B from west-central and southern regions dispersed west and stay at estuaries as the sea level rose about 18,000 years ago [60, 61]. The significant Fu’s *Fs* values of lineage A and B, MTR, TWR and JGE populations support above viewpoint (Table 1).

The Central Mountain Rang (CMR), composed of many mountain peaks above 3000 m in elevation, is an obvious geographical barrier for dispersal of freshwater species from west-to-east and vice versa in Taiwan [48, 62, 50, 52]. Haplotypes of the eastern population did not form a monophyletic group but were nested in west-central and southern populations, and some were distributed at the tip positions in lineages A (Figs 2 and 3). These tip haplotypes may represent recently occurring polymorphisms [63, 64]. Therefore, the eastern populations should not be considered endemic populations and are likely to have low contemporary gene flow across the CMR. The eastern population may be introduced via human activities. Human introduction of freshwater organisms was also found in freshwater fishes [4, 48], a freshwater crab [49], the bamboo viper [65] and fresh prawn [52] in Taiwan.

## Conclusions

Our study indicated a high level of genetic structure among the oriental river prawn populations in Taiwan. Two main lineages (A and B) were found, and lineage A was older than lineage B. The west-central and southwestern Taiwan and estuaries were mixing areas of these two lineages. According to genealogic analysis, the dispersal route of this species was from China to west-central and southern Taiwan, and then the part individuals belonging to lineage A and B dispersed southerly and northerly, respectively. And then part individuals in western coasts fell back to and stay at estuaries as the sea level rose. Demographic analyses implied a population expansion occurred during the recent history of the species.

## Supporting Information

S1 Dataset All combined sequences of 16S rRNA and COI of *Macrobrachium nipponense* and *M*. *asperulum* from Taiwan used in this paper. (FASTA)

S2 Dataset All sequences of 16S rRNA of *Macrobrachium nipponense* and *M*. *asperulum* from Taiwan used in this paper. (FASTA)

S3 Dataset All sequences of COI of *Macrobrachium nipponense* and *M*. *asperulum* from Taiwan used in this paper. (FASTA)
